# Reliable *In Silico* Identification of Sequence Polymorphisms and Their Application for Extending the Genetic Map of Sugar Beet (*Beta vulgaris*)

**DOI:** 10.1371/journal.pone.0110113

**Published:** 2014-10-10

**Authors:** Daniela Holtgräwe, Thomas Rosleff Sörensen, Prisca Viehöver, Jessica Schneider, Britta Schulz, Dietrich Borchardt, Thomas Kraft, Heinz Himmelbauer, Bernd Weisshaar

**Affiliations:** 1 CeBiTec & Department of Biology, University of Bielefeld, Bielefeld, Germany; 2 Molecular Breeding Sugarbeet, KWS Saat AG, Einbeck, Germany; 3 Syngenta Seeds AB, Landskrona, Sweden; 4 Max Planck Institute for Molecular Genetics, Berlin, Germany; 5 Centre for Genomic Regulation, Barcelona, Spain; National Institute of Plant Genome Research (NIPGR), India

## Abstract

Molecular markers are a highly valuable tool for creating genetic maps. Like in many other crops, sugar beet (*Beta vulgaris* L.) breeding is increasingly supported by the application of such genetic markers. Single nucleotide polymorphism (SNP) based markers have a high potential for automated analysis and high-throughput genotyping. We developed a bioinformatics workflow that uses Sanger and 2nd-generation sequence data for detection, evaluation and verification of new transcript-associated SNPs from sugar beet. RNAseq data from one parent of an established mapping population were produced by 454-FLX sequencing and compared to Sanger ESTs derived from the other parent. The workflow established for SNP detection considers the quality values of both types of reads, provides polymorphic alignments as well as selection criteria for reliable SNP detection and allows painless generation of new genetic markers within genes. We obtained a total of 14,323 genic SNPs and InDels. According to empirically optimised settings for the quality parameters, we classified these SNPs into four usability categories. Validation of a subset of the *in silico* detected SNPs by genotyping the mapping population indicated a high success rate of the SNP detection. Finally, a total of 307 new markers were integrated with existing data into a new genetic map of sugar beet which offers improved resolution and the integration of terminal markers.

## Introduction

The biennial plant sugar beet is a member of the order *Caryophyllales* and is grown commercially for sugar production mainly in the temperate climate zones. Currently, about one quarter of the world's sugar production is derived from sugar beet. The plant is not only grown for table sugar production, it is also of increasing importance for production of bioethanol as a source of renewable energy [1], [2].

Sugar beet is a diploid allogamous crop in nature with 18 chromosomes (1n = 9) and an estimated haploid genome size of about 731 Mbp [3], [4]. During the last decade sugar beet was target of several genetic mapping approaches [3], [5]. A single nucleotide polymorphisms (SNP) based genome-wide association map addressing six agronomic traits has been published in 2011 [6]. Shortly after, a genetic map that had been tightly linked to a physical map in BACs was made available [7], as well as the first sugar beet reference transcriptome based on RNAseq data [8]. Recently, genome sequence assemblies from five double haploid sugar beet lines were published, including the high-quality genome sequence of the reference genotype KWS2320 [3]. This reference assembly comprises 566.6 Mbp and displays a N50 size of 1,7 Mbp.

In the past, sugar beet breeding companies as well as academic research institutes have spent considerable effort to build large segregating populations. The goals are, among others, the identification of quantitative trait loci (QTL) with agronomical relevance or fine mapping important monogenic traits, e.g. disease resistance. Positional cloning of genes and development of markers with improved diagnostic value, both aided by the availability of SNPs and genome sequence, will help to optimise the sugar beet breeding process and will speed up the development of new varieties.

SNPs are the most abundant type of DNA variation currently used as genetic markers, because of their suitability for automated detection and multi-parallel analysis. This allows high-throughput analyses of many markers and individuals [9]. Empirical evaluation and comparison of different marker systems revealed a good success rate for SNP marker in diversity analysis of sugar beet hybrid varieties [10]. Also, 2nd- generation sequencing technologies have enhanced genome-wide SNP discovery in crop plants [11]. However, a bottleneck for the discovery of valuable SNPs in small to medium large datasets is the reliability of polymorphic site detection. Therefore, either very large sequence datasets or sequences read information with a high reliability are applied. Since both options require a considerable effort in money and time, the exploitation of existing resources like large EST collections from Sanger technology is still meaningful. Such Sanger ESTs offer a long read length, helping to overcome problems caused by e.g. the error-prone assembly of cDNA sequences encoding highly conserved protein domains. In general, the assembly of transcriptome data from short RNAseq sequences possesses a significant bioinformatics challenge [12].

Over the last few years different strategies and pipelines for automated SNP discovery from large sequence datasets have been developed, e.g. PolyBayes [13], AutoSNP [14] and QualitySNP [15]. Some strategies for SNP detection make use of trace or quality files, for example the PHRED/PHRAP/PolyBayes system [13], [16]. AutoSNP and QualitySNP have shown to be useful for extracting reliable SNPs from EST sequence datasets where quality information is missing. Several pipeline packages for SNP discovery from 2nd-generation sequencing datasets have been described [17]–[19], among these *CASAVA* (Consensus Assessment of Sequence and Variation, Illumina) and the *Probabilistic Variant Caller* (included in the commercially available CLC software packages, CLC Bio, Denmark). These SNP identification pipelines are e.g. advancements of the PolyBayes pipeline [17]. The annotation-based SNP detection package AGSNP [19] allows the use of all current types of 2nd-generation sequencing reads under the assumption that at least one of them generates relatively long reads.

In this study we describe a strategy for the identification, evaluation and verification of reliable polymorphisms between conventional Sanger ESTs and 454-FLX ESTs, by making use of the quality values for both sequence types. One purpose of this work was to explore the potential of combining existing, reliable and high-quality sequence datasets with the power of cost-effective, high-throughput sequence generation. Furthermore, we used the SNPs and InDels identified for generation of new genetic markers and an extended genetic map of sugar beet.

## Material and Methods

### Plant material and DNA

For RNA isolation, plants of the K1P2 parent of the KWS1 mapping population were grown in the greenhouse under long day conditions on soil for seven weeks. Reduction of photosynthetic/chloroplastic gene expression was performed by etiolation for four days prior to harvest. Subsequently, leaf tissue was collected in the dark, frozen in liquid N_2_ and stored at −80°C until use.

For SNP genotyping by amplicon sequencing genomic DNA was extracted from the parents K1P1 and K1P2 of the KWS1 mapping population, the K1F1 genotype as well as the F2 genotypes of the KWS1 mapping population. Genomic DNA was preperated from leaf material with the modified CTAB-DNA extraction method as described in Rosso *et al*. [20]. The KWS1 population was provided by KWS SAAT AG and had 183 F2 individuals, as described in [7]. All F2 plants were derived from a single F1 clonal plant. K1P1 is identical to the DH line KWS2320 that represents the sequenced genotype [3], parent K1P2 is an inbred line that contains about 10% remaining heterozygousity.

### RNA extraction and cDNA synthesis

Total RNA from frozen leaf tissue was extracted using the Qiagen Plant RNAeasy Kit. The tissue was ground under liquid nitrogen, and RNA was extracted using the RLT buffer provided with the kit. The RNA obtained was treated with Ambion DNA-free DNase and subsequently quantified using a NanoDrop2000c (Thermo Fisher). The total RNA amount per 100 mg tissue was 37.54 µg. For cDNA synthesis using oligo-dT priming the SuperScript-II RT Kit (life technologies) was used. The complete cDNA was used for 454-FLX library construction and sequencing.

### 454-FLX EST generation

Preparation and sequencing of the 454-FLX sequencing library was performed according to the manufacturer's instructions (GS FLX General library preparation kit/emPCR kit/sequencing kit [21]). About 6.5 µg of sugar beet cDNA was shorn by nebulisation and size selected by AMPure Beads (Agencourt) to 300–700 base fragments. These fragments were used to construct a single-stranded shotgun library that was used as template for single-molecule PCR. The amplified template beads were recovered after emulsion breaking and selective enrichment. The GS FLX was run for 220 cycles of four solutions containing either dTTP, dATP, dCTP and dGTP reagents. The raw reads of the 454-FLX run are available from SRA (sequence read archive, accession number SRX647739).

### Processing of 454 reads

The sequences of sugar beet K1P2 cDNA from the Roche 454-FLX system were assembled using the GS Assembler (version 2.6) from Roche Applied Science to generate 454-FLX EST data. The software filters reads for contaminations and low quality bases, and it keeps the quality scores. Short reads (<80 nt) and repeats were removed. The assembly was performed with default settings. For simplicity we do not differentiate between contigs, isocontigs and isogroups. We refer to each continuous DNA sequence that has been assembled from overlapping cDNA reads and regardless of its length as a contig.

### Pre-processing of Sanger reads

The Sanger sequence data used in this study have been published [22], are derived from the genotype K1P1 and are available from GenBank/EBI (dbEST). The original tracefiles were newly basecalled using the program *phred* (version 0.020425.c [23]). The obtained sequences were filtered for slippage using the algorithm of Telles *et al*. [24]. Removal of sequences related to *Escherichia coli* K12 (GenBank No. NC_000913) was done using BLASTN [25] (*blastall* version 2.2.24). Reads with *E. coli* matches displaying a BLAST e-value lower than 1e-40 were excluded from further evaluation. After masking vector sequences with *cross_match* (version 0.990329, [26]) and filtering of low quality bases (minimum average quality score 20) by a sliding window approach (window size 50 nt), the longest unmasked subsequence was taken as high quality sequence (HQS), if this was longer than 50 nt. All Sanger HQS were assembled by using *phrap* (version 0.990329, [26]).

### Polymorphic site discovery

From the K1P1 Sanger EST assembly as well as from the K1P2 454-FLX ESTs only contigs and singlets larger than 200 bp were used for polymorphic site detection. Matching K1P1 and K1P2 contigs and singlets were detected with *blastall* and aligned with *MAFFT* (version 6.857b, default parameters) [27]. The resulting alignments were grouped into three classes. First, monomorphic alignments; these do not contribute to the results and were removed. Second, alignments with an identity below 97% were considered as potentially not reliable due to generation of false-positive results; these were removed as well. Third, the remaining alignments which were further evaluated. The Python program *mmfind* (available from BiBiServ [28]) was written, optimised and used to evaluate polymorphic alignments based on the quality values for each 454-FLX and Sanger EST position. *mmfind* detects mismatches in fasta-formatted sequence alignments and is able to handle ambiguity codes as well as associated quality scores of different kinds of sequences. Additionally, *mmfind* trims alignments according to specified parameters (e.g. minimal sequence quality or minimal number of aligned sequences at the ends), produces a consensus sequence and combines alignments. The following mismatch types are recognized by *mmfind*: one-base SNP, multi-base SNP, one-base InDel, multi-base InDel and mixed types.

### Scoring of polymorphisms

Classification of polymorphisms into the categories “good”, “usable”, “uncertain” and “bad” was performed based on three criteria. The neighbourhood quality standard (NQS, [19]) as the first criterion was considered ‘passed’ if the average score of the polymorphic bases was at least 20 and the average score of the five bases up- and downstream was at least 15 (test A). The second criterion was considered fulfilled (test B) if the minimal distance to the end of the alignment was larger than 80 bp and the third criterion was considered ‘passed’ (test C) if the length of the polymorphism was less than 3 bp. SNPs and InDels that passed all three criteria were classified as “good”. Passing test A and/or test B but failing test C leads to the category “useable”. Succeeding criterion C but failure of criterion A and/or criterion B leads to the category “uncertain”, whereas SNPs and InDels that do not pass any criteria were recognized as “bad”.

To locate the new marker coordinates within RefBeet1.2, sequence tags flanking the polymorphic sites (100 nt upstream and 100 nt downstream) for each of the markers were aligned to the reference sequence using BLASTN, and by considering always the best hit. The individual SNP positions were calculated from the resulting alignments with an in-house script.

### SNP validation, amplicon sequencing and segregation analysis

For SNP validation and/or marker value determination by amplicon sequencing, primer pairs were designed to fit the flanking sequence of the addressed SNP using the Primer3 software [29] with an average length of 22 nucleotides and a melting temperature around 58°C. Using knowledge gained from already sequenced and annotated genomes like *Arabidopsis thaliana*, poplar (*Populus trichocarpa*) and rice (*Oryza sativa*), predictions of the exon/intron borders on the cDNA sequences and the likely intron sizes were performed. Deduced from these predictions an expected amplimer size of 500 to 1000 bp on genomic DNA was targeted. Genomic DNA (gDNA) from relevant sugar beet genotypes was used as template in PCR reactions with each primer pair under the following conditions. Each 20 µl PCR reaction contained 2 ng gDNA, 0.5 µM of each primer, 200 µM of each dNTP, 0.5 U *Taq* polymerase and reaction buffer with 10 mMTris/HCl (pH 8.0), 50 mM KCl and 2.5 mM MgCl_2_. PCR was performed at 96°C for 2 min, followed by 40 cycles at 96°C for 30 s, 58°C for 30 s, 72°C for 30 s and a final extension at 72°C for 3 min. Amplicons were visualised on agarose gels, purified with Exo-SAP IT (USB Corporation, Ohio, USA) and sequenced in both directions using the ABI Prism BigDye Terminator Cycle Sequencing kit (Applied Biosystems, Foster City, CA, USA) on an ABI Prism 3730xl sequencer (Applied Biosystems, Foster City, CA, USA). Sequences of the two parental lines and from the F1 generation were assembled to a consensus sequence for each genotype, aligned with the 454-FLX-Sanger-EST reference alignment sequence and evaluated for the primarily predicted SNPs as well as for additional polymorphisms using the Sequencher version 4.9 sequence analysis software [30].

For the segregation analysis based on Sanger technology the same verified primer pairs were used for amplicon generation and sequencing. The obtained sequence reads for each F2 individual were assembled into a multiple alignment and evaluated for segregation using the ABH code. An A stands for the homozygous alleles from the K1P1, B for the alleles from the K1P2 and H for the heterozygous allele combination. Primers for KASPr-marker assays were designed using a tool provided by KBiosciences based on the SNP locus sequence.

### Genetic mapping

The raw data of the new markers were systematically cleaned through a pre- and post-mapping diagnosis according to Jansen *et al.* [31]. Genotypes with at least 10% missing values and outstanding large number of recombinations for each chromosome were discarded. Two or more markers showing no recombination at a locus were treated as a single locus. Four markers were excluded due to dominance or distorted segregation. The processed marker data from this study together with marker data from BeetMap [7] and the genotyping data of the terminal markers [32] were grouped in *JoinMap 4* [33] at independence LOD of 3 to 37. Linkage groups were assigned to chromosomes according to [34]. Mapping was done in a two step approach: the marker order per chromosome was determined with RECORD [35] using the following parameters: 30 cM maximal gap size, 0.1 as maximal fraction of extra recombinations allowed and 5 steps to reach upper limit. The resulting marker order was used as fixed order when calculating marker distances in JoinMap. Here the Haldane mapping function was used with the mapping parameters of the maximum likelihood mapping algorithm as described in Dohm *et al.* [7]. Maps were drawn with MapChart [36].

## Results and Discussion

### 454-FLX EST generation

A Roche 454-FLX run analysing cDNA from leaves of sugar beetgenotype K1P2 generated a dataset of about 61 Mbp which was composed of 266,666 single reads. The mean read length was 227 nt. After elimination of short reads (below 50 nt), approximately 70% of all reads were aligned and assembled into 5,716 contigs (minimal length 200 bp, average length of 670 bp), summing up to a total contig length of about 3.83 Mbp.

**Figure 1:**
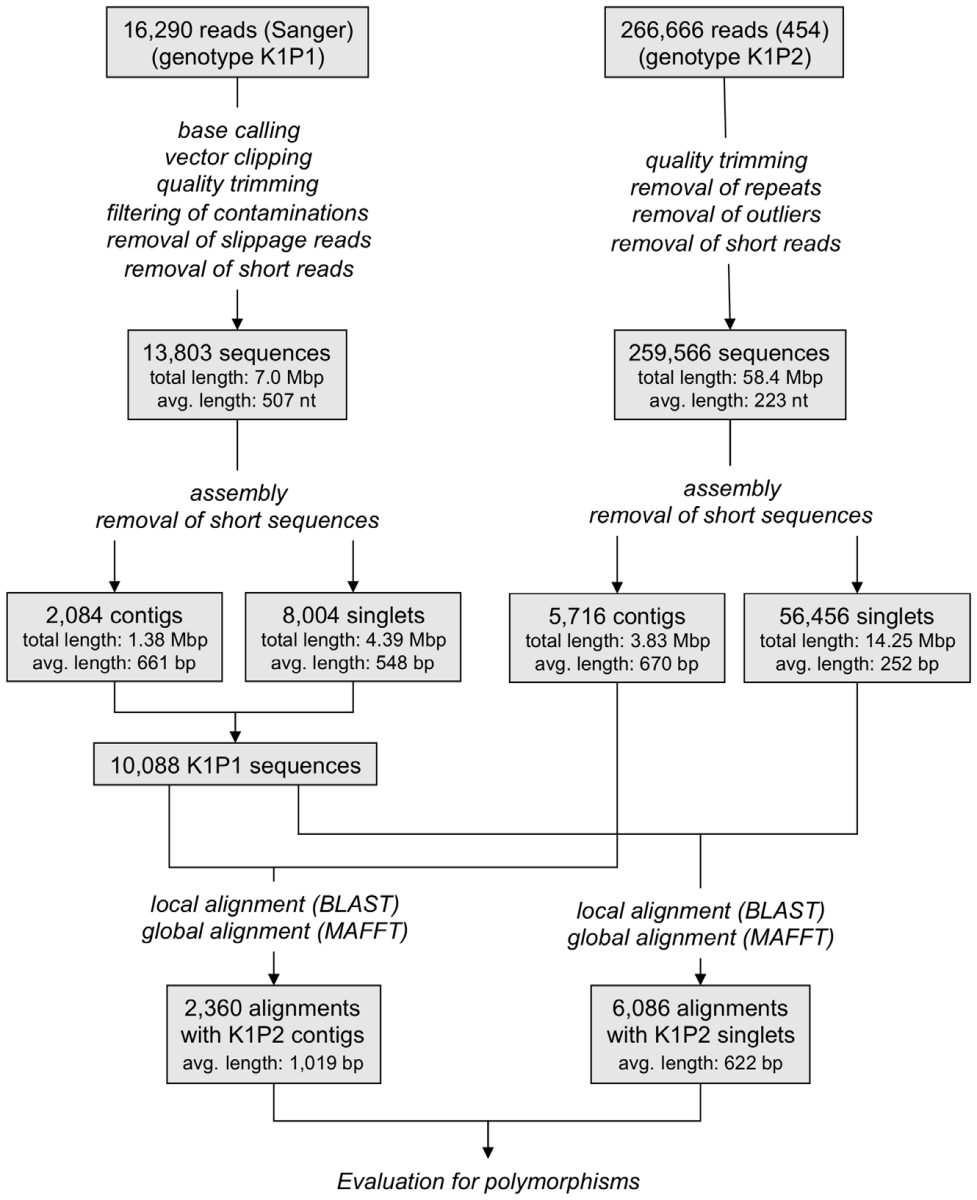
Workflow of data processing for polymorphic site detection. The analysis steps (marked [1] to [9]) executed from the two starting data sets to the polymorphic alignments are summarised.

The remaining 56,456 single reads (singlets) with at least 200 nt and a mean read length of 252 nt accounted for 14.25 Mbp in total. The two datasets were analysed in parallel to identify polymorphisms also in low expressed genes. From 16,290 Sanger EST reads derived from genotype K1P1 (see Material and Methods), 2,084 contigs and 8,004 singlets were generated. Inspection via BLASTN [25] revealed that unwanted sequences like chloroplast, mitochondrial, known repeat sequences and bacterial contamination (in total less than 1%) were not a concern. An overview of the input data and the processing steps is depicted in Figure 1.

For 5,471 (54.2%) of the 10,088 unique sugar beet Sanger ESTs [22] a homolog was detected in the complete 454-FLX dataset. Valid matches were filtered by setting the minimum length to 200 nt and the identity to at least 97%. A total of 2,278 ESTs matched only to 454-FLX contigs, and 3,193 only to 454-FLX singlets, while 160 ESTs matched sequences of both types.

### Polymorphic alignments

Sequences of the K1P1/K1P2 homologs were aligned to produce one alignment for each non-overlapping sequence pair. This resulted in 8,446 individual alignments in total, 2,360 for the 454-FLX contigs and 6,086 for the 454-FLX singlets (Figure 1). For simplicity, these will be referred to as K1P2-contig or K1P2-singlet alignments. As a consequence of the global alignment using MAFFT, that included also alignments of several 454-FLX contigs or singlets to one Sanger EST, some alignments showed a rate of sequence divergence of more than 3%. In addition, about 12% of the K1P2-contig based alignments and 21% of the K1P2-singlets based alignments were found to be monomorphic. Finally, 1,394 (59%) of all K1P2-contig based and 3,302 (54%) of the K1P2-singlet based sequence alignments were considered as reliable polymorphic alignments (Table 1) that belong to 2,265 loci (clusters of K1P1 and K1P2 sequences, combining for example 5′ and 3′ Sanger ESTs from the same gene).

**Table 1:** Evaluation of alignments for polymorphic site detection.

		K1P1-sequences aligned to K1P2-sequences					
		contig alignments		singlet alignments		total	
[1]	total number alignments	2,360	100%	6,086	100%	8,446	100%
	total length (bp)	2,407,018	100%	3,789,889	100%	6,196,907	100%
	avg. length (bp)	1,019		622		734	
[2]	alignments too polymorphic	692	29%	1,503	25%	2,195	26%
	total length (bp)	776,042		932,616		1,708,658	
[3]	monomorphic alignments	274	12%	1,281	21%	1555	18%
	total length (bp)	243,577		799,965		1,043,542	
[4]	polymorphic alignments	1,394	59%	3,302	54%	4,696	56%
	total length (bp)	1,387,399		2,057,308		3,444,707	
[5]	polymorphisms	6,472		7,870		14,323*	
[6]	total SNPs	5,759		6,298		12,057	
	single base SNPs	4,580		5,091			
	multibase SNPs	1,179		1,207			
	avg. length multibase SNPs (bp)	2.8		2.4			
[7]	total InDels	704		1,562		2,266	
	single base InDels	551		1,399			
	multibase InDels	153		163			
	avg. length multibase InDels (bp)	20.9		3.8			
[8]	polymorphic bases raw in [3] & [4]	11,675		10,031		21,706	
[9]	frequency of polymorphisms in [3] & [4]	1/139		1/285		1/207	

*Excluding 19 cases with uncertain mismatch typing due to lower sequence quality.

Alignments between Sanger sequence reads (K1P1) and either K1P2-singlets or K1P2-contigs were created and evaluated with regard to the presence and type of polymorphic sites. Numbers in brackets reflect successive analysis steps.

From comparative BAC sequencing [37] and multiple EST sequence alignments [38] it has been deduced that sugar beet haplotypes differ within exon regions around 1% (1.4%) at the nucleotide level. Making use of the comparably long Sanger sequencing and 454-FLX derived ESTs and the application of the 3% divergence limit allowed a good identification of paralogous sequences thereby minimizing the rate of false positive polymorphism calls. This long sequence based SNP detection approach is in particular suited to identify multiple SNPs in a small region as well as InDels of medium size. Comparable results regarding these issues could be expected if Illumina derived RNAseq data were applied to a transcriptome assembly and later used for polymorphic site detection.

### Polymorphic site detection

Detection of mismatches within the alignments was performed with the newly implemented Python program *mmfind*, which is able to deal with the quality scores from Sanger and 454 sequencing. The detection of polymorphic sites by *mmfind* has proven to be fast and convenient for commonly formatted alignments. By applying *mmfind* to the fasta-formatted alignments, a total of 14,342 mismatches were identified and evaluated. The 1,394 K1P2-contig based alignments yielded 6,472 putative polymorphisms, while the evaluation of 3,302 K1P2-singlet based alignments resulted in 7,870 putative polymorphisms. In 19 cases (nine for K1P2-contig alignments and ten for K1P2-singlet alignments) the mismatch typing was uncertain due to low sequence quality. These cases were rejected, resulting in 14,323 sequence polymorphisms that were used for subsequent analyses and statistics. The results are summarised in Table 1, including statistics for single base and multibase SNPs as well as for InDels.

Even after discarding high polymorphic alignments with more than 3% mismatches, there was a considerable difference in frequency of putative polymorphisms between K1P2-contig (1 mismatch in 139 bp) and K1P2-singlet (1 mismatch in 285 bp) alignments. By evaluating the data of both alignment types, 21,706 polymorphic raw base positions in about 4.49 Mbp were observed, leading to an overall SNP frequency of 1/207 bp. This overall observed SNP frequency of 1/207 bp is above the frequency of 1/324 bp published earlier. Reason for this could be that former polymorphic site detection was based on only one Sanger derived sequence of limited length for each parent of the mapping population increasing the probability to miss or discard notable SNPs at both borders. Due to this reason the real SNP frequency may be slightly higher than our observed SNP frequency of 1/285 bp if only the singlet-based alignments were processed. This frequency is comparable to SNP rates in coding regions of e.g. 1/200 bp in barley [39] and 1/191 bp in soybean [40].

### Polymorphic site classification and statistics

The detected mismatches were validated for suitability and reliability using strict parameters. Our evaluation for suitability addresses technical aspects of marker development, as there is the requirement of at least about 80 adjacent bases around the polymorphism for primer design and a maximum of three consecutive polymorphic bases. The reliability was evaluated by the Neighbourhood Quality Standard (NQS, [19], [41]). Mismatches suitable in terms of the technical aspects and reliable according to the NQS quality check were categorised as “good” polymorphisms. The other categories comprise: “usable” (not suitable, but reliable), “uncertain” (suitable, but not reliable) and “bad” (not suitable and not reliable). In total 6,510 (45.5%) mismatches were classified as “good”, 2,992 (20.9%) as “usable”, 4,770 (33.3%) as “uncertain” and 51 (0.4%) as “bad” (Table 2). The “good” mismatches cover 1,562 (69.0%) of all 2,265 represented loci. It should be noted that polymorphisms in the category “usable” may easily become useful if sufficient genomic sequence information becomes available for the locus in question.

**Table 2:** Scoring of polymorphisms.

	SNPs (incl. multi-base)	InDels	SNPs & InDels	Loci (best status)	Category
Total	12,057	2,266	14,323	2,265	
All tests succeeded	5,424	1,086	6,510	1,562	“good”
Test A succeeded, test B and/or C failed	2,529	463	2,992	291	“usable”
Test A failed, test B and/or C succeeded	4,055	715	4770	410	“uncertain”
All tests failed	49	2	51	2	“bad”

Polymorphic sites were categorised according to three criteria as described in the methods section. Test A: Neighbourhood Quality Standard, average score of polymorphic bases> = 20, average score of 5 bases up-/downstream> = 15; test B: minimal distance to border> = 80 bp; test C: polymorphism length < = 3 bp.

### Elimination of already known loci

Former approaches for SNP marker development have partly consulted the same Sanger EST dataset that has also been used in this study. The Sanger ESTs contributed to a small amount to the markers generated for two SNP-based genetic maps, namely the map produced by K. Schneider [38] and the BeetMap [7]. Since the polymorphic sites discovered in this work should also contribute to the development of new genic SNP markers for sugar beet, the elimination of polymorphisms at loci for that markers already exist was essential. We investigated the 2,265 loci with new polymorphisms (see Table 2), and excluded 94 loci because they were already covered with markers. For the majority of these loci (88) an EST-based marker already existed, whereas in six cases a BAC end sequence-based marker was identified. It is important to note that a given locus can be addressed with different polymorphisms. Therefore, the SNPs assayed to genotype the 94 loci are not necessarily included in the alignments generated in this study.

To use the repeated detection of already validated polymorphisms as a proof of the reliability of our strategy, we filtered all known markers for those that addressed SNPs located in exons and identified 68 such cases. In the approach presented here, we have detected SNPs between Sanger and 454 ESTs which have to be located in exons while many of the other genic markers addressed polymorphisms in introns. Then, we checked if these 68 exonic SNP positions are covered by one the 4,496 polymorphic alignments. Only 21 SNP positions addressed by known markers were part of one of the polymorphic alignments. Of these 21, 19 were classified as “good”, one as “uncertain” and one other represented a false negative case. These results indicated at least satisfying reliability of our strategy.

### Marker development and SNP-genotyping

SNPs were established as the most abundant co-dominant marker class and used as efficient and robust marker systems [42]. Besides this, polymorphisms or mutations in coding DNA regions (cSNPs) may lead to changes in the amino acid sequences and affect gene function. Therefore, cSNPs are highly valuable for marker development.

Our SNP identification was first evaluated at the level of genomic DNA using 20 candidate SNPs classified as “good” and 23 classified as “useful”. These candidate SNPs were selected after prediction of intron positions (see Material and Methods). All of the 20 chosen candidate SNPs from the category “good” could be confirmed by Sanger amplicon sequencing of K1P1, K1P2 and the F1 from which the KWS1 mapping population was derived. In contrast, only nine of the 23 “useful” SNPs were confirmed. For this evaluation only true single base SNPs were used, because Sanger sequence analysis of InDels could potentially be problematic for the heterozygous F1 genotype. In addition, six of the identified and verified genic SNPs (three of each category) were used successfully as genetic markers which were scored by Sanger sequencing in KWS1.

After this first small scale pilot study had demonstrated the high quality and reliability of the strategy for polymorphic site detection, 325 polymorphisms (282 of the category “good” and 43 of the category “uncertain”) were used for marker development using the KASPar technology [43]. Genotyping platforms like KASPar/melting curve require a significant amount of sequence information around the tracked SNP to work properly. Mostly at least 30–50 nucleotides on each site are necessary, which is easily reached for the majority of SNPs detected by the approach described here. For the same reason no SNPs from the category “usable” were chosen. Finally, 225 polymorphisms were genotyped in the KWS1 population with a success rate of about 69%. The “good” mismatches again displayed a higher success rate for marker development than “uncertain” ones: of 282 “good” mismatches 211 (75%) were successfully genotyped, whereas only 14 of the 43 “uncertain” mismatches (33%) yielded results (Table 3). The markers were designated with the code “EBS” followed by a 4-digit number, e.g. ‘EBS0278’. The polymorphic sequences (Table S1), the primer sequences (Table S2) as well as the scoring data (Table S3) are provided as data supplements. Taken together, the computational as well as wet lab effort and costs were in relation to the success rate of reliable SNP detection and genetic mapping of coding SNPs very advantageous.

**Table 3:** Detailed results of the marker generation.

	All tests positive		Failed NQS	
Selected and screened polymorphisms	282	100%	43	100%
Successful genotyped polymorphisms	211	75%	14	33%
Converted to markers	199	71%	14	33%

All tests comprises test A: Neighbourhood Quality Standard; test B: minimal distance to border and test C: polymorphism length. Neighbourhood Quality Standard (NQS) is a possible criterion to evaluate the reliability. More details can be found in Materials and Methods and Table 2.

### Generation of an extended and improved genetic map of sugar beet

Genotyping of the newly generated markers in the KWS1 population revealed the expected segregation ratios and independence between the targeted loci. By applying JoinMap 4 [33] all 215 new EST-derived markers could be integrated into the former genetic map (BeetMap) of Dohm *et al.* [7] resulting in an improved genetic map, designated BeetMap-3 (Table 4; Figure S1). The observed random distribution of these markers on all nine linkage groups of sugar beet gave strong evidence for the high reliability of the polymorphic site detection and processing approach.

**Table 4:** Comparison of marker number and genetic distance between genetic maps.

	previous genetic map (BeetMap)		current genetic map (BeetMap-3)		marker number comparison	
Chr.	cM	# marker	cM	# marker	added	deleted
1	93.2	85	103.0	111	28	2
2	87.2	82	130.2	114	32	0
3	107.5	89	116.2	112	25	2
4	108.0	86	123.6	115	30	1
5	101.8	145	147.8	199	54	0
6	102.7	147	137.9	181	34	0
7	101.9	132	146.6	171	39	0
8	92.5	90	110.5	119	29	0
9	92.0	127	125.6	163	36	0
**Sum**	**886.8**	**983**	**1141.4**	**1285**	**307**	**5**

Total marker numbers and genetic distances (cM) are presented for each of the nine sugar beet chromosomes for the previous published [7] and for the current map constructed within this study. Added and deleted markers are itemised separately. Added markers belong to this study, deleted markers are from the previous BeetMap.

Choosing EST sequences as a basis for polymorphic site detection in sugar beet improved the current genetic map [7] especially by integrating markers in coding regions. BeetMap-3 enlarges the number of EST derived markers from 283 to 497. Taking into account the very good success rate of more than 75% for SNP genotyping if using polymorphisms from the category “good” and the generation of polymorphic alignments for more than 1,500 loci, we generate at least 1,100 genetically distinct potential marker locations, just by adding a small FLX-EST data set to the already published Sanger ESTs. If a genotyping platform allows the evaluation of InDels larger than 3 bp or needs less than 30 bp sequences on both sides of the tracked polymorphism, also mismatches of the category “useful” are promising candidates in addition the “good” ones. The usage of polymorphisms from the category “uncertain” is as expected not favourable, but it offers options to find a marker for a gene of interest that is otherwise not covered.

**Figure 2:**
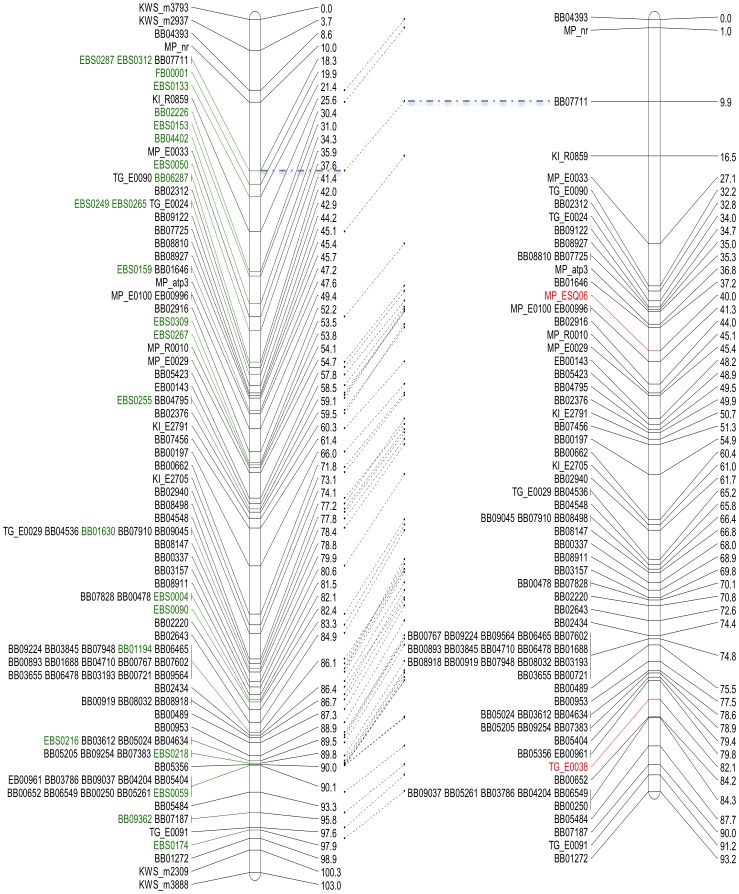
Display of chromosome 1 comparing the current and former genetic map derived from the KWS1 mapping population. The new map designated BeetMap-3 is shown on the left, the former BeetMap on the right. Names of markers added by this study are highlighted in green, excluded markers are marked in red. Terminal marker were named by using the prefix “KWS_”. Cosegregating markers are indicated by identical map positions.

Before starting to calculate a new genetic map, we set out to gather data from additional markers that might be beneficial for extending the marker set for the KWS1 population. First, 31 markers corresponding to BACs that mark the chromosome termini [32] were determined in the KWS1 population. Second, 61 validated BAC-based markers from a study on marker recovery from BAC end sequences that were also determined in the KWS1 population were added. In total, the number of genetic markers was increased from 983 to 1285 markers in BeetMap-3. A total of 307 newly identified genetic markers were integrated, while only five markers from the former map [7] had to be removed due to the stringent criteria for putative linkage. For each chromosome, between 25 and 54 new markers accounting for distinct loci were integrated. The total map length increased from 886 centi-Morgan (cM) in BeetMap [7] to 1141 cM in BeetMap-3 (Table 4). A comparison of the two maps of chromosome 1 demonstrated high co-linearity for the overall marker order (Figure 2). Data on the other chromosomes are provided in Figure S1. This extension of the marker set for sugar beet map construction led to a significant and important increase of marker density as well as map coverage. Very good co-linearity of BeetMap-3 with BeetMap was observed for all chromosomes, demonstrating the high quality of both maps. Besides the enhanced map coverage by the new EST-derived markers, the integration of endpoint markers led to a moderate map inflation.

### SNP distribution in the genome

The positions of the SNP markers were determined within the RefBeet reference assembly [3] (see Material and Methods). All SNPs with a single exeption were identified (see Table S4). The exception is the EST-derived SNP EBS0280_193, and the failure in detecting this SNP is most probably due to the fact that the reference assembly covers the sugar beet genome to 96% in terms of genes, which is very good but not 100% [3].

As validated by genetic mapping, the newly detected SNPs are randomly distributed throughout the reference assembly. Also, in almost all cases the genetic marker order is co-linear with the physical order of scaffold sequences which further verifies the high accuracy of the reference assembly. Out of the 276 additional marker sequences, 25 hit chromosomally assigned but genetically unanchored scaffolds. In addition, 11 marker sequences hit the chromosomally unassigned part of the assembly. The genetic mapping information of these 36 markers could be used to genetically assign the respective scaffold and contig sequences. However, we refrain from reordering the current reference assembly at this stage for two reasons. First, only for a single unanchored scaffold (Bvchr8_un.sca002) markers with a genetic distance larger than 0.5 cM were detected that allow orienting the newly anchored sequence relative to the north/south ends of the chromosome; for all other scaffolds and contigs there is no hint regarding their correct orientation. Second, the potential improvement reached by assigning a few additional sequences to the reference assembly is not worth the hassle of creating an updated assembly that affects all position-related information like e.g. gene names.

## Conclusions

The strategy established for a reliable *in silico* identification of polymorphic sites by combining 454-FLX and Sanger reads, and the successful application of the categorisation of polymorphic sites for marker development resulted in three main achievements. First, the analysis strategy described here can be transferred to other species. Second, the categorisation and the established filter criteria allow a promising conversation rate between the initially detected sequence polymorphisms and the finally scored markers. Third, the work presented has resulted in a significantly improved genetic map of sugar beet with higher resolution and integrated terminal markers. Although the 454 technology runs out of date, the same strategy can be applied to medium or long size RNAseq data generated by e.g. the Illumina MiSeq platform and assemblers that keep the quality values.

## Supporting Information

Figure S1 **Updated genetic map displaying all nine chromosomes, layout and naming identical to** **Figure 2** **.**
(PDF)

Table S1 **Marker names and polymorphic sequences.** Mismatches are given in brackets, starting with the K1P1 allele.
(XLSX)

Table S2 **Marker assay data. Marker types and primer sequences.**
(XLSX)

Table S3 **Scoring data from new markers and F2 individuals for genetic mapping.** In the ABH table scoring the K1P1 allele leads to an A, the K1P2 allele corresponds to B and heterozygous calls lead to H.
(XLSX)

Table S4 **Position of SNPs of the newly added markers from BeetMap-3 on the contig and scaffold sequences of the reference sequence RefBeet1.2.**
(XLSX)
