# Supplementary figures and images for: Reliable In Silico Identification of Sequence Polymorphisms and Their Application for Extending the Genetic Map of Sugar Beet (Beta vulgaris)
Source: PLoS One. 2014 Oct 10;9(10):e110113. doi: 10.1371/journal.pone.0110113 (PMC4193868; doi:10.1371/journal.pone.0110113)

# New BeetMap-3 (left) old BeetMap (right)

1

1

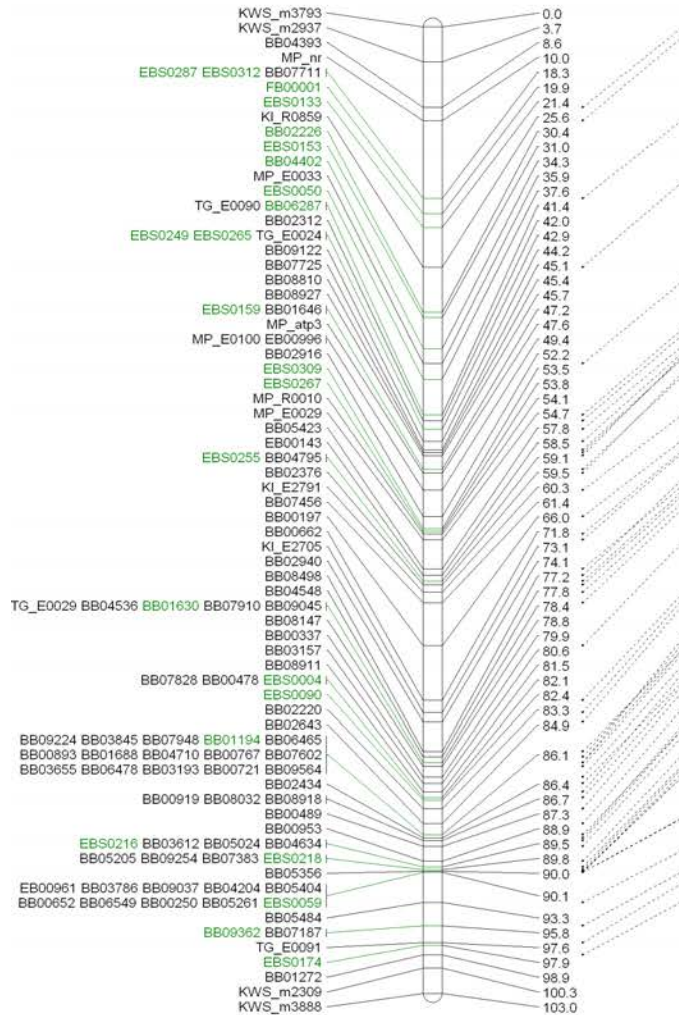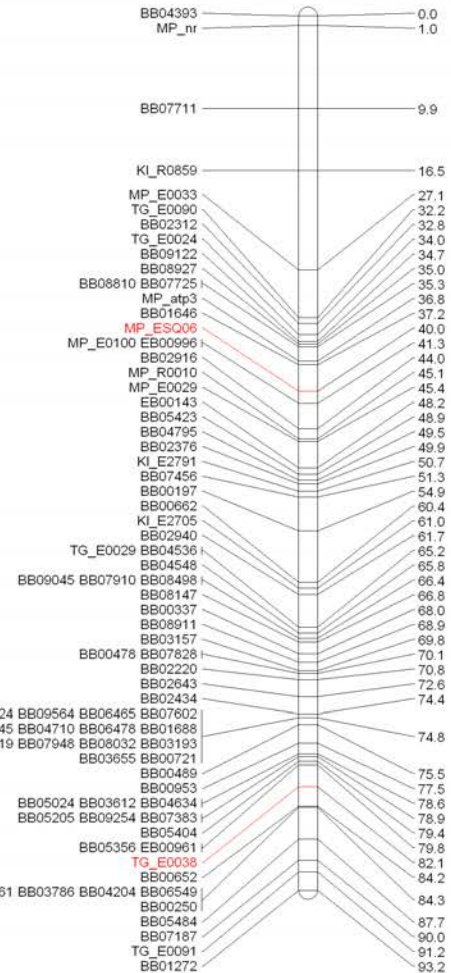

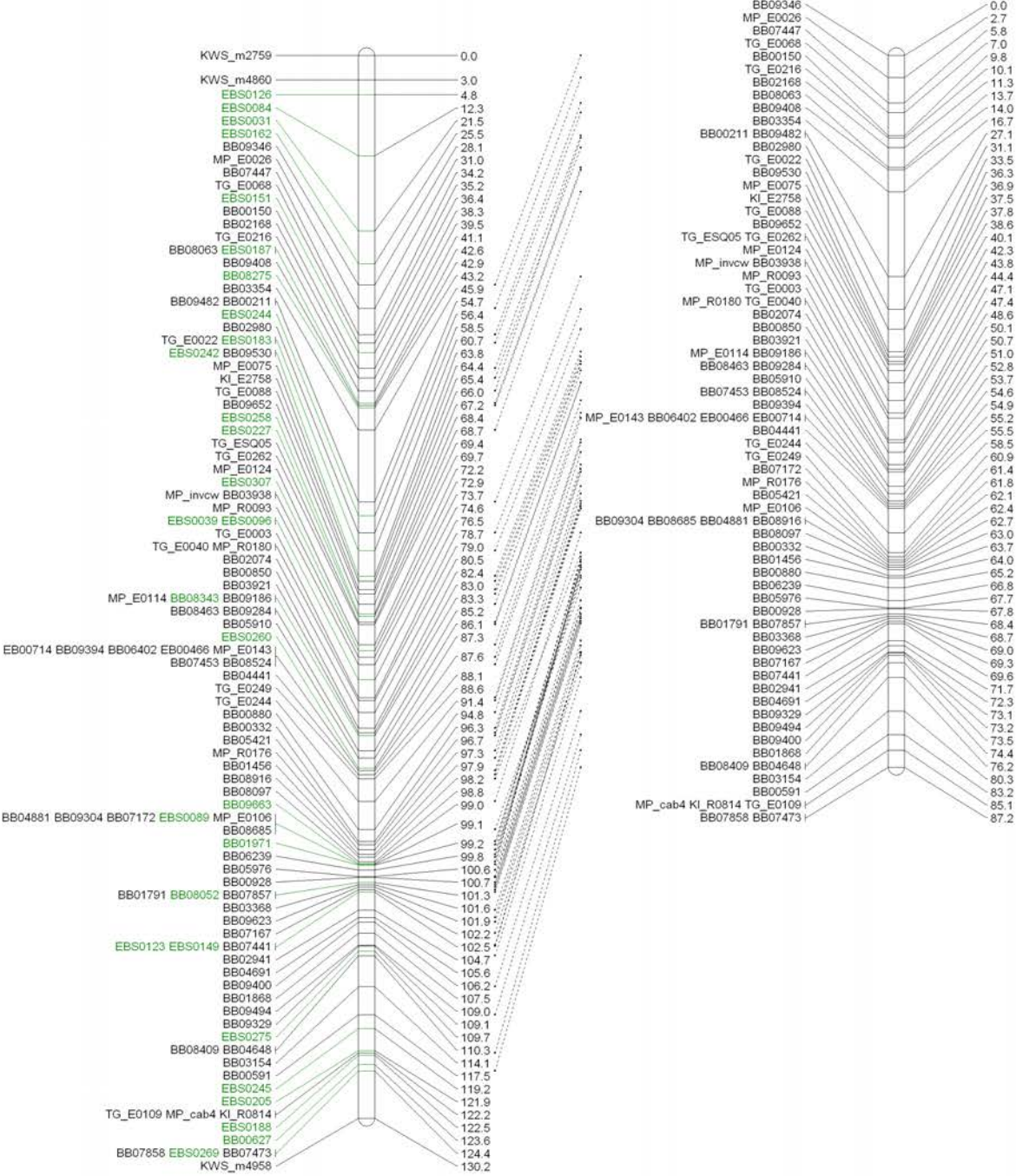

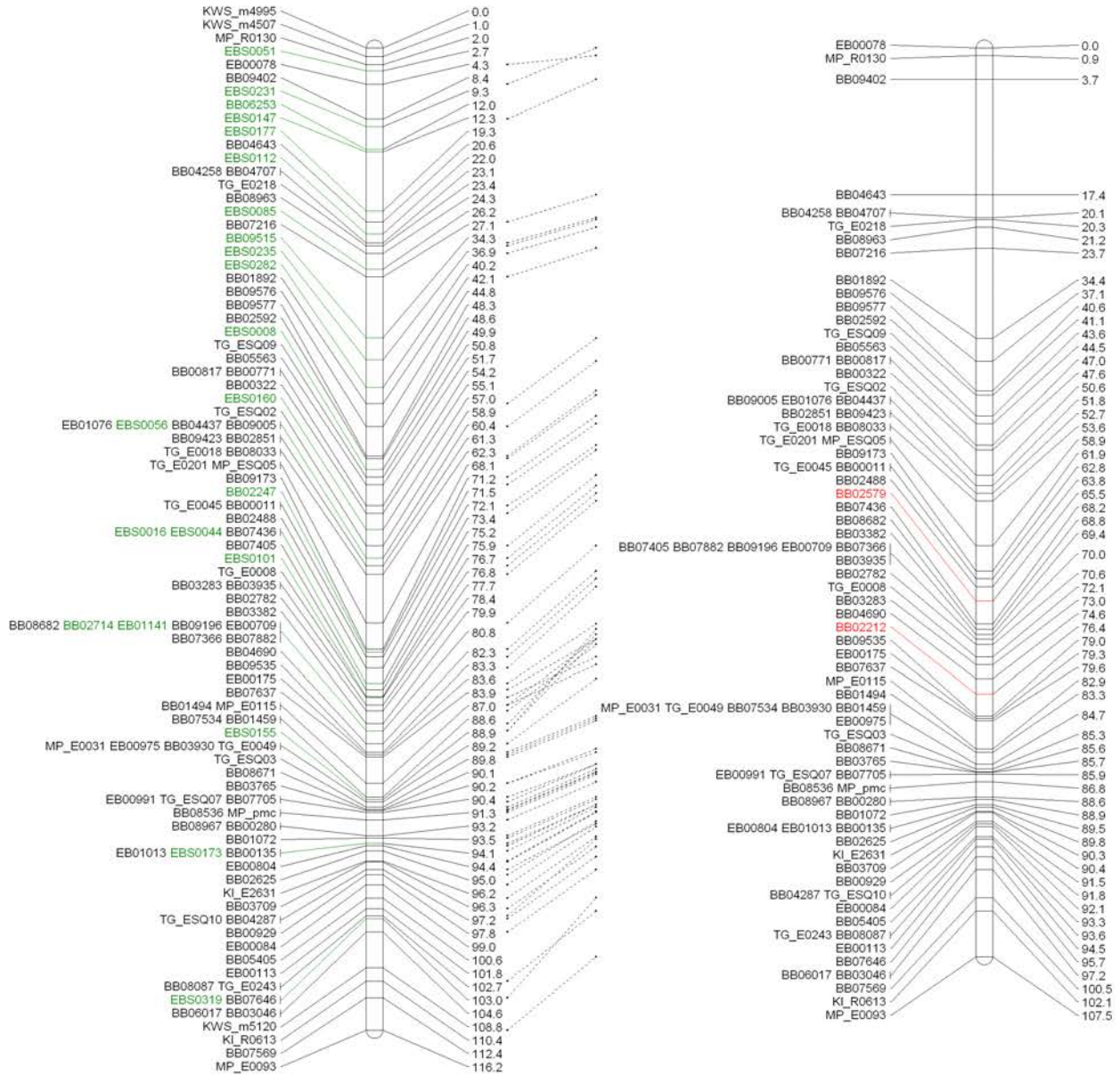

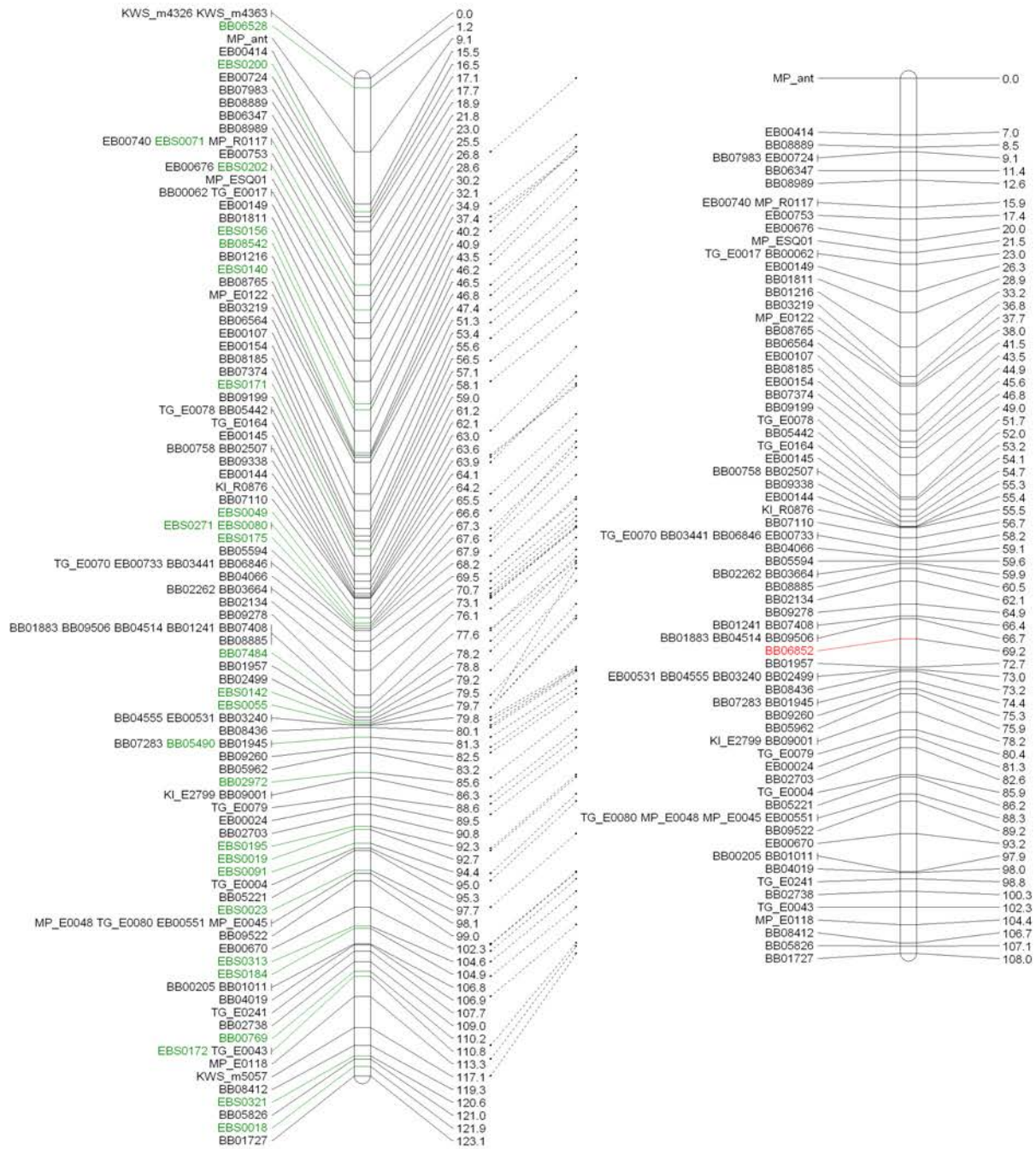

## New BeetMap-3 (left) old BeetMap (right)

5

5

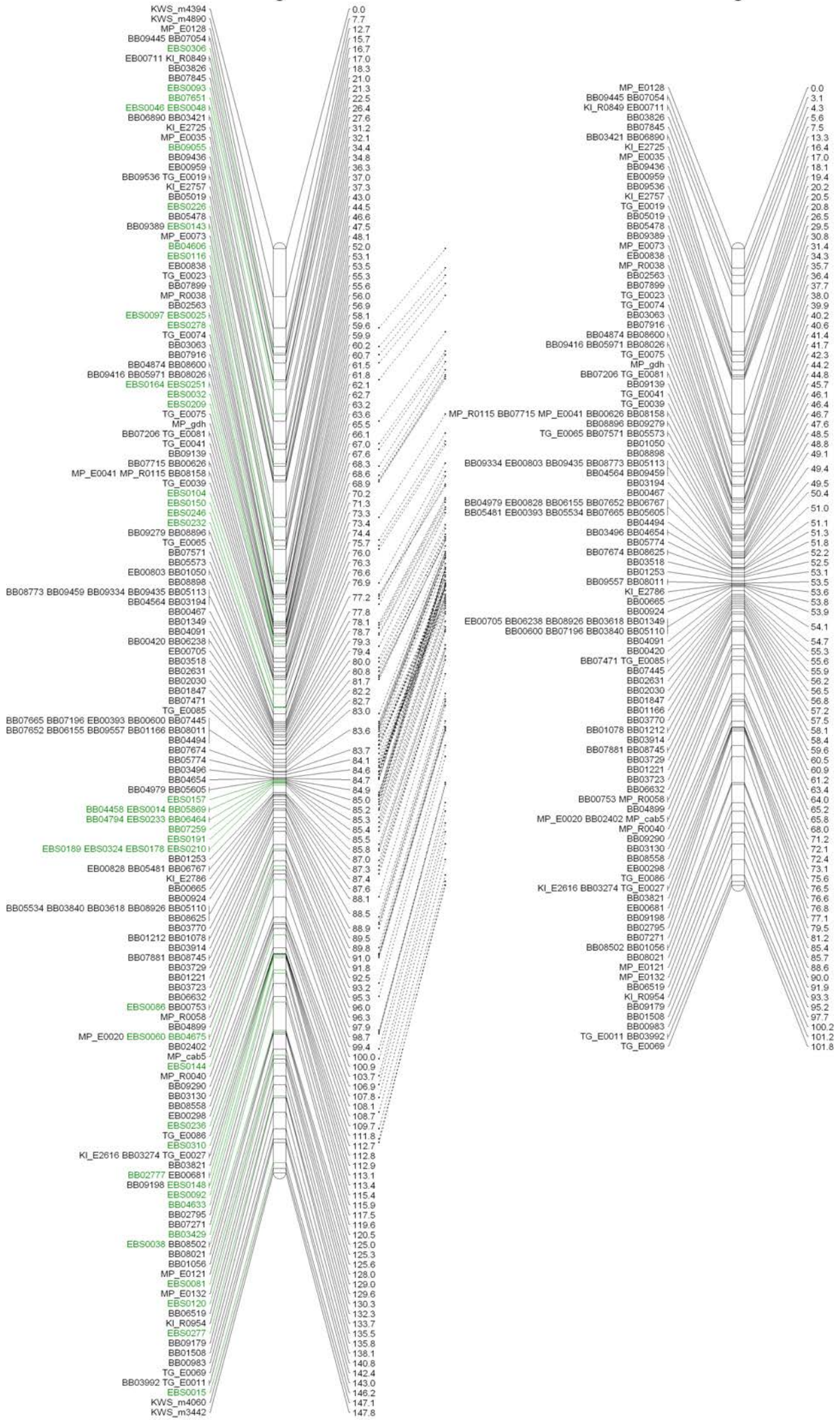

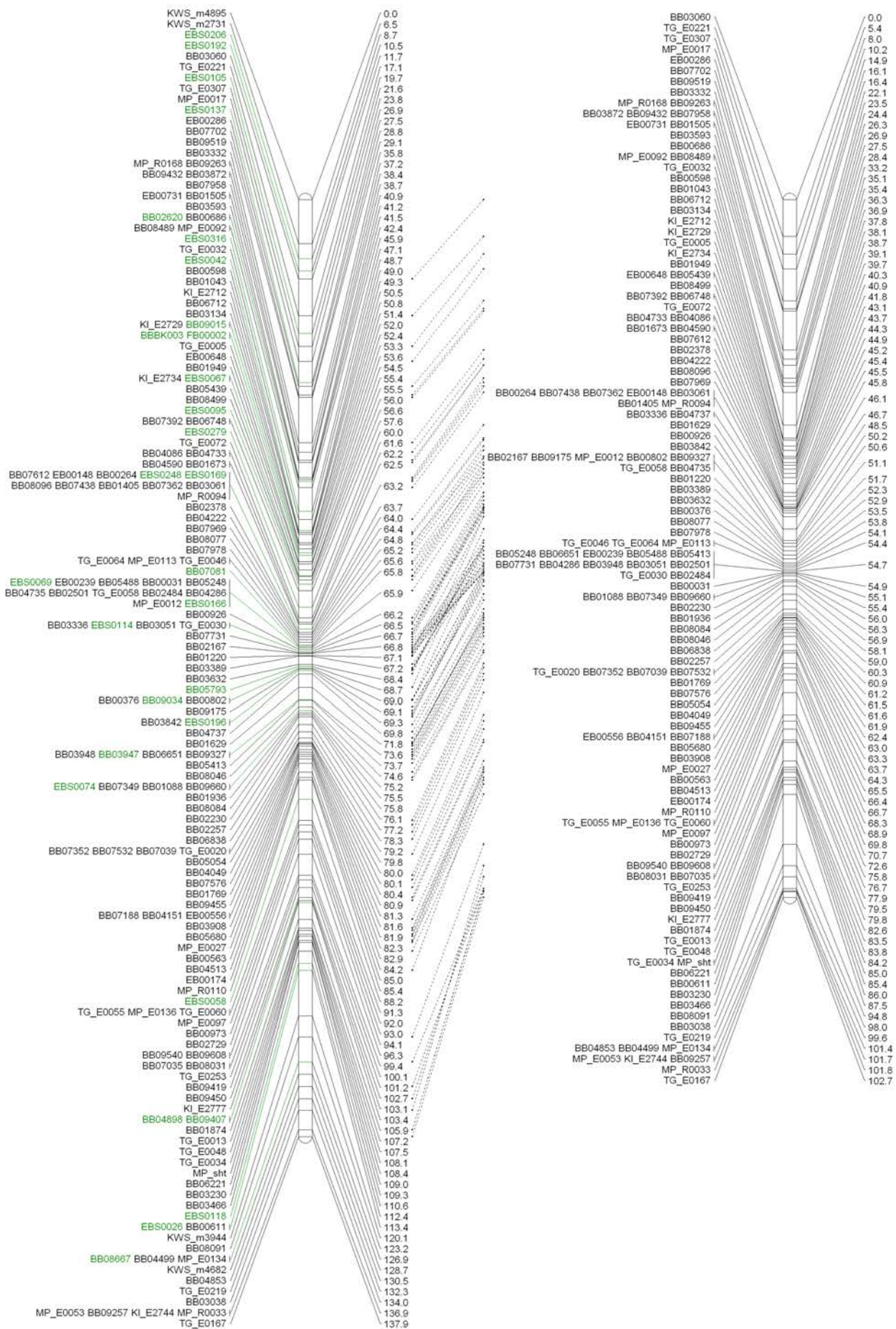

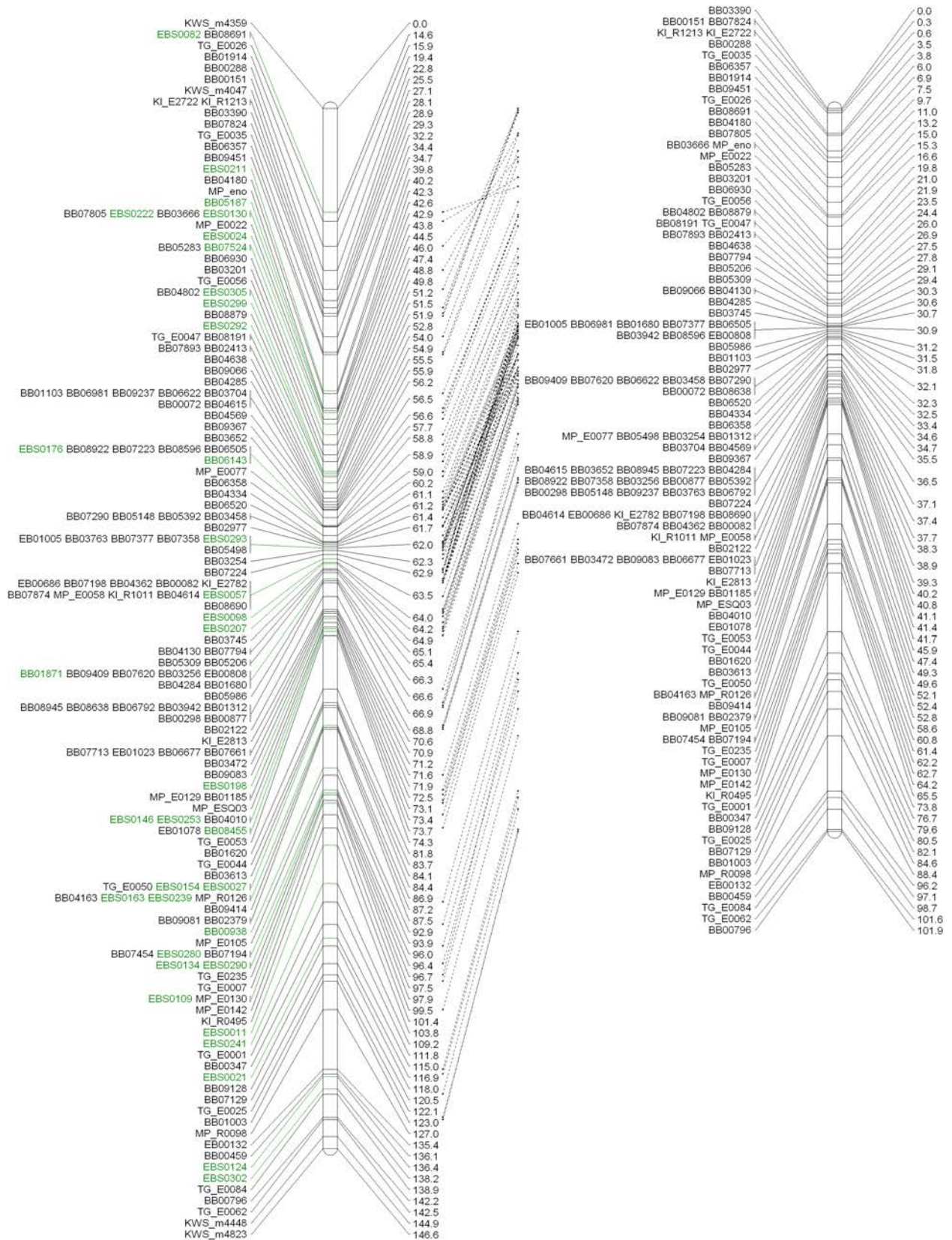

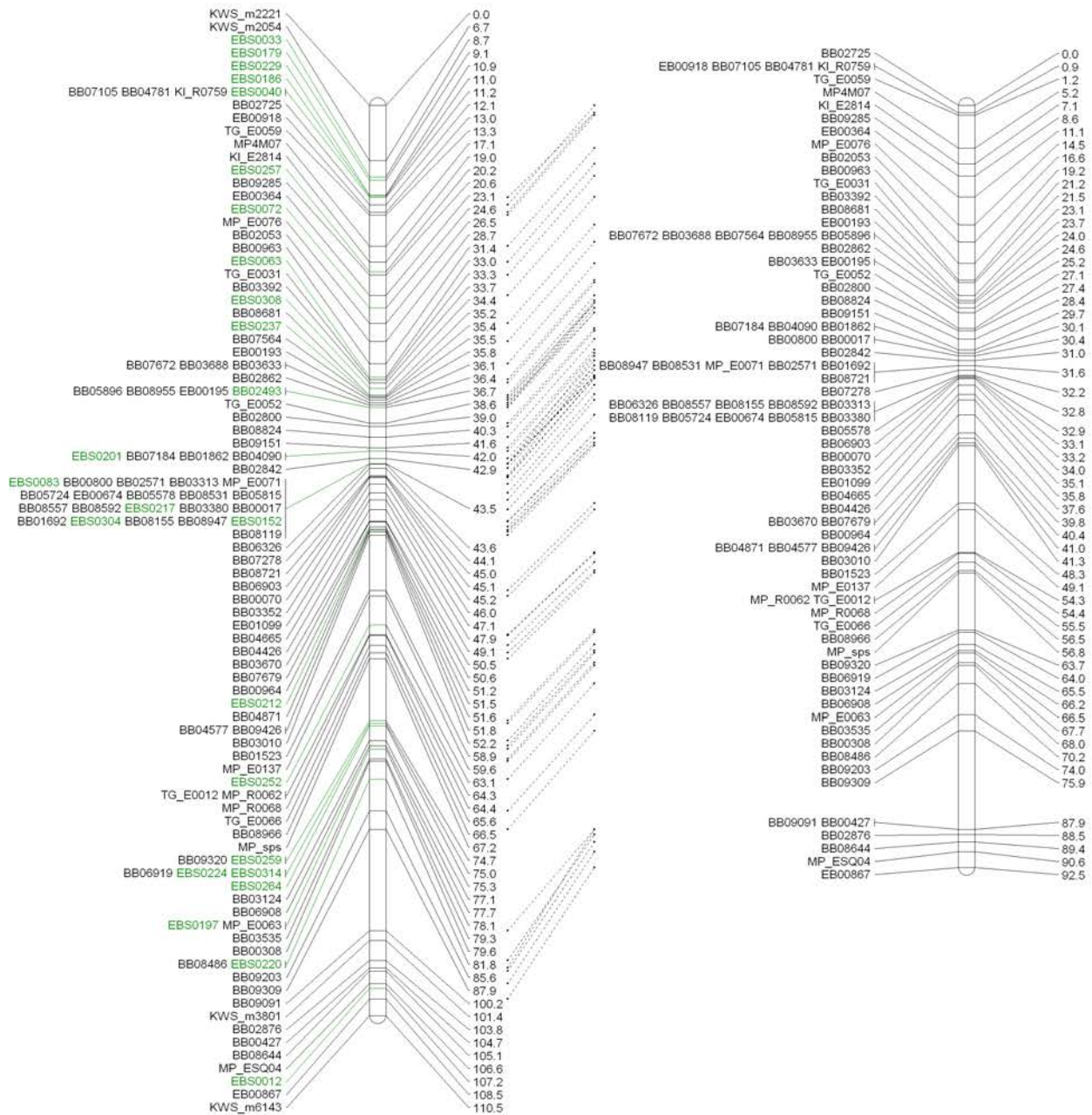

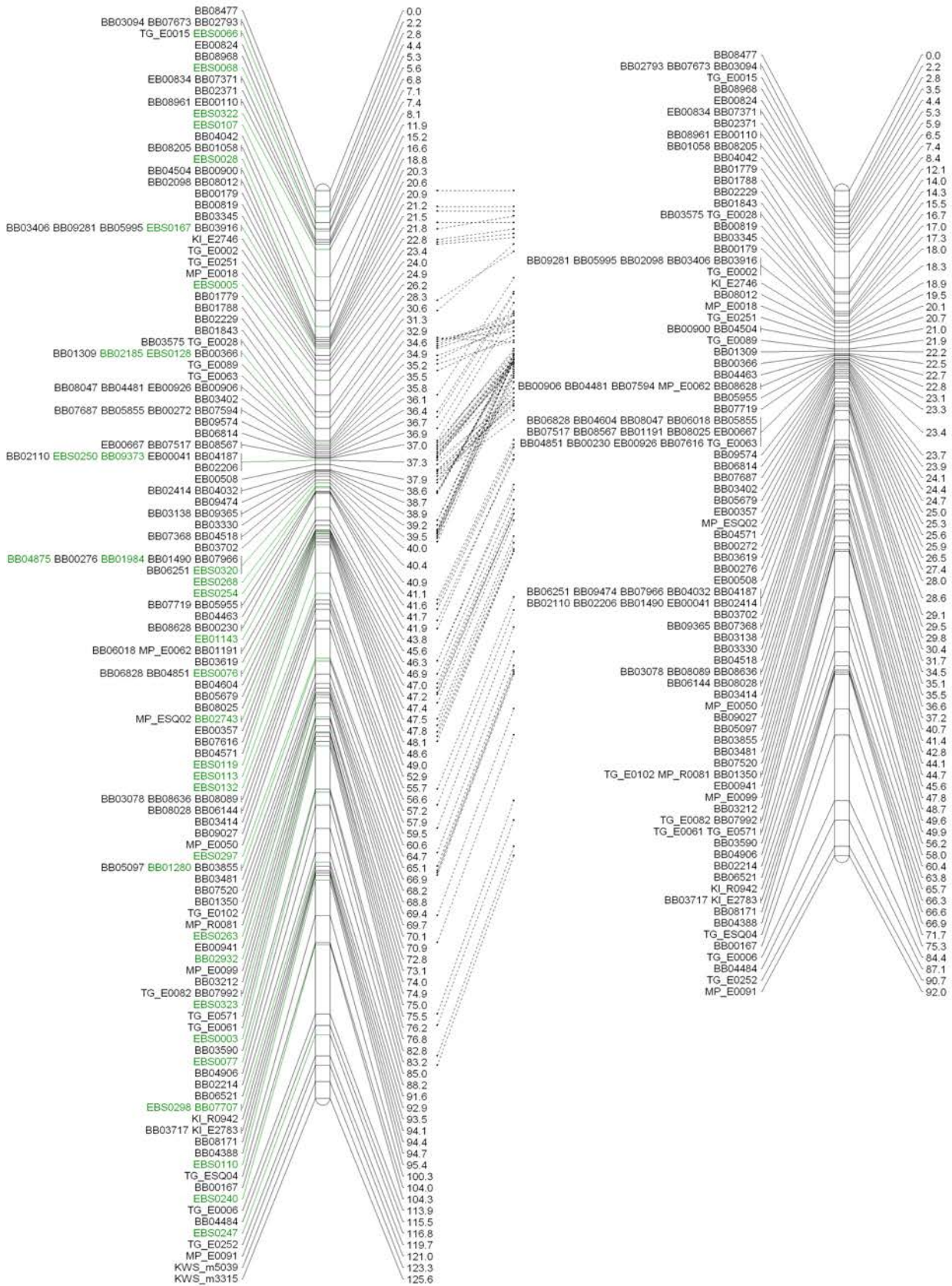

Supplement: Figure S1 — Updated genetic map displaying all nine chromosomes, layout and naming identical to Figure 2 . (PDF) [file pone.0110113.s001.pdf]
